# Conference on "Emerging Infectious Diseases: Meeting the Challenge".

**DOI:** 10.3201/eid0103.950309

**Published:** 1995

**Authors:** J M Hughes


### Conference on "Emerging Infectious Diseases: Meeting the Challenge"

Emerging infectious diseases, the leading cause of death worldwide, continue to pose difficult challenges to clinicians, public health professionals, and biomedical researchers in academic settings and industry. Addressing these challenges requires a cohesive effort to develop prevention strategies and to communicate them effectively to the health care community, the public, and policy makers.

On June 5-6, 1995, the New York Academy of Medicine and the New York State Department of Health convened to examine the problem of emerging infections. The speakers addressed four themes: 1) emerging infectious diseases: why and why now? 2) transmission of emerging infectious diseases: old modes, new agents; 3) surveillance and sentinel systems for infectious diseases; and 4) emerging infectious diseases: what is to be done?

The first three themes were addressed through presentations by 20 experts. The fourth was divided into six segments focusing on diagnosis, the role of the microbiology laboratory in surveillance, other surveillance issues, approaches to epidemic investigations, risk perception, and global issues.

Speakers consistently alluded to recent complacency about infectious diseases in the United States and stressed the need for the clinical, public health, and research communities to work with the biomedical industry in confronting emerging infectious disease challenges in this era of transition to managed health care. In his opening address Joshua Lederberg from Rockefeller University reminded participants that the struggle between humans and microbes could be characterized as a battle of "wits versus genes." Margaret Hamburg, Commissioner of the New York City Department of Health, emphasized that plague in India and Ebola virus infection in Zaire were reminders that the world is a global village, that considering domestic and international diseases as separate entities is an outmoded concept, and that many conditions that contribute to disease emergence or reemergence in the developing world are also present in the United States, adding to our domestic vulnerability to emerging infections.

Other speakers focused on the evolution of virulence, the molecular basis of pathogenesis, observations on factors contributing to the plague epidemic in India in 1994 and the Ebola outbreak in Zaire in 1976; foodborne and waterborne diseases; airborne diseases; zoonoses; sexually transmitted and bloodborne diseases and the increasing problem of antimicrobial resistance in both hospital and community settings. Concerns were expressed about the possibility of a "post-antimicrobial era" in which available drugs are no longer effective against common bacterial infections. Other speakers focused on innovative approaches to surveillance at the local, state, national, and international levels. James LeDuc from the World Health Organization (WHO) provided an update on the emerging infections resolution passed by the World Health Assembly in May 1995 and other WHO activities related to detecting and responding to emerging and reemerging diseases.

Among the themes recurring throughout the conference were the challenges that microbes will continue to pose; the critical role of the modern microbiology laboratory in detecting and responding to emerging and reemerging infections; and the limitations of existing capacity at the local, state, national, and international level to respond to these challenges. Human resource, equipment, diagnostic reagent, and facility needs were addressed, and resource needs were emphasized. Training needs of medical students, clinicians, epidemiologists, microbiologists, entomologists, mammalogists, behavioral scientists, and other researchers were also stressed. Additional emphasis was placed on the critical importance of communicating alerts about clusters of illness, data on disease trends, and guidelines for disease prevention; the need for educating professionals, the public, and policy makers about the critical importance of these issues; the need for strengthening existing partnerships and developing new ones, particularly with health maintenance organizations, the pharmaceutical industry, and nongovernmental organizations (including medical missionary organizations); and the need to carefully identify priorities.

Conference participants resounded the message of the 1992 Institute of Medicine Report, *Emerging Infections: Microbial Threats to Health in the United States*, "Pathogenic microbes can be resilient, dangerous foes. Although it is impossible to predict their individual emergence in time and place, we can be confident that new microbial diseases will emerge." Particular future concerns included a possible influenza pandemic, the emergence of vancomycin resistance in *Staphylococcus aureus*, the occurrence of large dengue hemorrhagic fever epidemics in the Western Hemisphere, and the likelihood that additional chronic diseases will be found to have infectious etiologies. Concerns were also expressed about the possibility of a terrorist incident involving an infectious agent and the potential difficulties in detecting and responding to such an episode.

The New York Academy of Medicine plans to use the discussions during the conference in formulating an agenda for further action.

**James M. Hughes**
National Center for Infectious Diseases
Centers for Disease Control and Prevention
Atlanta, Georgia, USA

### Japanese Encephalitis Acquired in Australia

Japanese encephalitis (JE), a mosquito-borne flaviviral disease of humans and animals, is a major public health problem in Asia, where an estimated 50,000 cases occur each year. There has been concern that the range of epidemic JE may be expanding.

On April 5, 1995, an outbreak of three cases of JE was recognized in Australia. Two of the cases were fatal; all were among residents of an island in Australia's Torres Strait, which lies between mainland Queensland and Papua New Guinea. JE was confirmed in two of the patients by polymerase chain reaction (Jeffrey Hanna, Queensland Health, pers. comm.). No other cases were reported. This is the first recognized episode of JE acquired in Australia.

Control activities on the Australian island began on April 7. The community was informed about the importance of personal mosquito protection measures. In addition, larvicides were applied, and areas were fogged to kill adult mosquitoes.

The patients were all male, aged 6 to 44 years. All were hospitalized with symptoms that included fever (up to 40ºC), stiff or painful neck, headache, and abdominal pain. Two patients were unconscious at the time of admission.

Acute-phase sera showed elevated JE virus immunoglobulin M (IgM) titers. Two of the patients also had detectable levels of Kunjin and Murray Valley encephalitis virus IgM, but the JE IgM titers were significantly higher in each case.

Flaviviruses have also been isolated from the sera of each of two asymptomatic island residents. Preliminary tests suggest that these are both JE virus. Blood taken from 10 horses and 12 domestic pigs living near humans on the island was also tested. All 12 pigs and 9 of the horses had high JE titers by hemagglutination inhibition assay. Neutralizing antibody to JE virus was detectable in all the pigs and in four of the horses tested to date.

Details of the index case are as follows: The patient, a 16-year-old male, was admitted to Thursday Island Hospital on March 22, 1995. He was unconscious and was responsive only to painful stimuli. His neck was stiff, and he showed a preference for moving his right side. His illness had begun 3 days before. The day before admission he complained of abdominal pain. This patient had been mildly mentally retarded since birth and occasionally had generalized seizures but was generally healthy. He was transferred to Cairns Base Hospital, where a cerebral CT scan showed a nonenhancing hypodense lesion in his posterior right basal ganglia.

He had a leukocytosis of $17.3 \times 10^9$L, neutrophils, $15.2 \times 10^9$. His cerebrospinal fluid contained 150 leukocytes/µl with a differential count of 50% polymorphs and 50% mononuclear cells.

He had a generalized seizure and 2 days after admission, required mechanical ventilation. He never regained consciousness and died on day 17 of hospitalization (April 8).

---

Adapted from Hanna J, Ritchie S, Loewenthal M, et al. Probable Japanese encephalitis acquired in the Torres Strait. Communicable Diseases Intelligence 1995;19:206-7.

### USPHS and IDSA Collaborate on Guidelines to Prevent Opportunistic Infections in HIV-Infected Persons

U.S. Public Health Service (USPHS)/Infectious Diseases Society of America (IDSA) Guidelines for Preventing Opportunistic Infections in HIV-Infected Persons will be published in an August 1995 supplement of *Clinical Infectious Diseases*. The guidelines, which are intended for health care providers, are the result of collaboration between the Centers for Disease Control and Prevention (CDC), the National Institutes of Health, IDSA, numerous federal and nonfederal organizations, community groups, and HIV-infected persons. The guidelines are endorsed by the American Academy of Pediatrics, the Infectious Diseases Society of Obstetrics and Gynecology, and the Society of Healthcare Epidemiologists of America. Jonathan E. Kaplan, M.D. (CDC), Henry Masur, M.D. (NIH), and King Holmes, M.D., Ph.D. (University of Washington), chaired the USPHS/IDSA Prevention of Opportunistic Infections Working Group and are guest editors of the *Clinical Infectious Diseases* supplement.

CDC initiated work on the guidelines in early 1994; meetings were held in Atlanta in June and September to discuss and refine the recommendations.

The USPHS/IDSA guidelines address 17 opportunistic infections from three angles: 1) preventing exposure to opportunistic pathogens (e.g., sexual, occupational, and environmental exposure as well as exposure through pets, food, water, and international travel); 2) preventing opportunistic disease by chemoprophylaxis and vaccination; and 3) preventing disease recurrence. In this document, new recommendations were made and earlier recommendations were updated. For example, new guidelines recommend that in nonemergency situations, cytomegalovirus (CMV)-seronegative HIV-infected persons who require blood transfusions receive only

